# Assessing Risk Factors for Prolonged Intensive Care Unit Stay After Surgery for Adult Congenital Heart Disease: A Study From a Lower-Middle-Income Country

**DOI:** 10.7759/cureus.35606

**Published:** 2023-02-28

**Authors:** Russell Seth Martins, Usama Waqar, Hussain Ahmed Raza, Muhammad Kamran Younis Memon, Saleem Akhtar

**Affiliations:** 1 Medical College, Aga Khan University, Karachi, PAK; 2 Pediatrics and Child Health, Liaquat National Hospital and Medical College, Karachi, PAK; 3 Pediatrics and Child Health, Aga Khan University, Karachi, PAK

**Keywords:** postoperative complications, intensive care, tetralogy of fallot, ventricular septal defect, atrial septal defect, adult congenital heart disease

## Abstract

Background

Prolonged post-surgery intensive care unit (ICU) stay for congenital heart disease (CHD) has been explored in the pediatric population. However, there is limited data for adult CHD (ACHD), also called grown-up congenital heart (GUCH) disease, especially in low-resource countries where intensive care beds are scarce. This study identifies factors associated with prolonged ICU stay following surgery for ACHD in Pakistan, a lower-middle-income country (LMIC).

Methods

This retrospective study included all adult patients (⩾18 years) who underwent cardiac surgery with cardiopulmonary bypass for their CHD from 2011-2016 at a tertiary-care private hospital in Pakistan. Prolonged ICU stay was defined as stay >6 days (75^th^ percentile). Regression analysis was used to explore risk factors of prolonged ICU stay.

Results

A total of 166 patients (53.6% males) with a mean age of 32.05 ± 12.11 years were included. Atrial septal defect repair was the most common surgery (42.2%). Most patients were categorized as Risk Adjustment for Congenital Heart Surgery 1 (RACHS-1) Category 1 (51.8%) and Category 2 (30.1%). Forty-three of 166 patients (25.9%) experienced prolonged ICU stay. Complications occurred in 38.6% of patients postoperatively, with the most common being acute kidney injury (29.5%). On multivariable logistic regression adjusted for age, gender, and RACHS-1 categories, intraoperative inotrope score, cardiopulmonary bypass time, aortic cross-clamp time duration of mechanical ventilation, and postoperative acute kidney injury (AKI) were associated with prolonged ICU stay.

Conclusion

Surgeons managing ACHD in LMICs must strive for shorter operative durations and the judicious use of intraoperative inotropes in addition to anticipating and promptly managing postoperative complications such as AKI, to minimize ICU stay in countries where intensive care beds are a scarce resource.

## Introduction

As a result of advances in early diagnosis, cardiac surgeries, interventional techniques, and critical care, more than 90% of children born with congenital heart disease (CHD) in developed countries survive into adulthood and a high proportion of surgeries for adult congenital heart disease (ACHD) comprises reoperations [1]. However, in developing countries such as Pakistan, inadequate diagnostic techniques and management of CHD results in a large burden of CHD persisting into adulthood remaining undiagnosed and unrepaired [2].

Surgery for ACHD is associated with several possible postoperative complications, including chest infection, arrhythmias, wound infection, and pericardial effusion [3], which may require prolonged management in the intensive care unit (ICU). Prolonged ICU stay is an especially important outcome of surgery for ACHD, as beds are a scarce resource in many ICUs, particularly in resource-constrained countries like Pakistan. Prolonged ICU stays may reduce bed availability, lead to the cancellation of elective surgeries, and extend waiting times for other patients, which can adversely affect their outcomes [4-6], in addition to increasing resource utilization [7,8]. Moreover, prolonged ICU stay increases the risk of nosocomial infections, complications, and mortality for postoperative patients [5]. Prolonged ICU stays are also associated with excessive costs and burdens on patients and their families [9]. These factors necessitate efficient distribution and utilization of ICU resources [10], which require an understanding of the various preoperative, intraoperative, and postoperative factors associated with prolonged ICU stay after surgery for CHD.

Predictors of prolonged ICU stay after surgery for CHD have been explored in the pediatric population, and include factors such as age at surgery, gender, comorbid congestive heart failure, aortic cross-clamp time (XCT), cardiopulmonary bypass time (CPBT), length of surgery, type of procedure, and postoperative complications [11-14]. However, similar data for adult patients undergoing surgery for ACHD is limited overall, and virtually non-existent from lower-middle-income countries (LMICs). Understanding factors causing prolonged ICU stay after surgery for ACHD in Pakistan will provide insight into ways of reducing ICU stay and maximizing scarce ICU resources. Our aim was to explore risk factors of prolonged ICU stay among patients undergoing surgery for ACHD. 

This article was previously presented as a meeting abstract at the Society of Thoracic Surgeons (STS) 17th Annual Perioperative and Critical Care Conference on September 26, 2020.

## Materials and methods

Study setting

This retrospective study was conducted at the Aga Khan University Hospital (AKUH), Karachi, Pakistan. The AKUH is an academic center accredited with the Joint Commission International (JCI) and has a capacity of 700 hospital beds. Demographic, clinical, and diagnostic information is archived in an in-house computerized data system at AKUH in a retrievable form.

Inclusion criteria

All adult (≥ 18 years) patients who underwent cardiac surgery with cardiopulmonary bypass (CPB) for their congenital heart diseases from January 2011 to December 2016 at AKUH were included in this study. These surgeries included atrial septal defect (ASD) closure, ventricular septal defect (VSD) closure, Tetralogy of Fallot (TOF) repair, aortic valve (AV) repair, mitral valve repair, pulmonary valve replacement, aortic coarctation repair on cardiopulmonary bypass, and Fontan operation. Patients with combination lesions and those undergoing redo surgeries were also included.

Exclusion criteria

Patients who underwent cardiac surgeries for acquired cardiac diseases, those undergoing off-pump surgeries, and those requiring thoracotomy were excluded.

Procedures

Nonprobability consecutive sampling was used for all patients falling under the inclusion criteria. Data was collected from the computerized data system and cross-checked with patient medical records using a standardized proforma. Preoperative characteristics included age, gender, body mass index (BMI), primary diagnosis, comorbidities, and history of previous surgery. The Risk Adjustment for Congenital Heart Surgery 1 (RACHS-1) scoring system was used to classify patients into risk categories according to the procedures they underwent [15]. Intraoperative variables included type of congenital heart disease repair (univentricular vs biventricular), CPBT, XCT, and vasoactive inotrope score (dopamine dose [μg/kg/min] + dobutamine dose [μg/kg/min] + 100 × epinephrine dose [μg/kg/min] + 10 × milrinone dose [μg/kg/min] + 10,000 × vasopressin dose [unit/kg/min] + 100 × norepinephrine dose [μg/kg/min]). Postoperatively, recorded variables included occurrence of any postoperative complications including AKI defined according to the Kidney Disease Improving Global Outcomes (KDIGO) criteria [16], duration of mechanical ventilation, duration of ICU stay, and total length of hospital stay.

At our setting, ACHD patients are extubated via a standard cardiac surgery extubation protocol that encompasses: (a) neurological goals (alertness and ability to follow commands, head lift > 5 seconds, ability to grip hands and move all extremities, and intact gag reflex), (b) hemodynamic goals (systolic blood pressure > 90 mmHg or < 160 mmHg, sinus rhythm with 60-100 beats per minute, no ischemic changes or sustained arrhythmias, cardiac index > 2.1, and chest tube drainage < 70 ml/hour for two consecutive hours), and (c) respiratory/oxygenation goals (fraction of inspired oxygen < 50%, positive end-expiratory pressure of about 5 cm water, oxygen saturation > 92%, respiratory rate < 26 breaths per minute, arterial pH > 7.35, partial pressure of carbon dioxide in arterial blood (PaCO_2_) 35-45 mmHg, arterial oxygen pressure (PaO_2_) >70 mmHg, negative inspiratory force < -25 cm water, and tidal volume > 0.5 ml/kg). Patients are transitioned out of the ICU when they are vitally stable with no or minimum requirement of inotropes following extubation and mobilization.

Primary endpoint

There are no universal criteria to define prolonged ICU stay. Previous studies have used > 72 hours and > 96 hours as cut-offs depending upon their 70^th^ and 75^th^ percentiles [17-19]. We defined prolonged length of ICU stay as postoperative ICU stay longer than the 75^th^ percentile of the overall cohort, which classified prolonged ICU stay as any ICU stay > 6 days.

Statistical analysis

The IBM SPSS Statistics for Windows, Version 21.0 (2012; IBM Corp., Armonk, New York, United States) was used to analyze data. Descriptive statistics were presented as mean ± standard deviation and frequencies (%) for continuous and categorical variables, respectively. Independent sample t-tests were used for the comparison of means and Chi-squared tests for the comparison of percentages. Univariate and multivariable logistic regression models (adjusted for age, gender, and RACHS-1 category) were used to establish the relationship of preoperative, intraoperative, and postoperative variables with prolonged ICU stay. Odds ratio (OR), 95% confidence interval (CI), and p-values were presented in tables. A p-value < 0.05 was considered statistically significant.

## Results

Demographics and preoperative characteristics

This study included 166 patients, the majority of whom were males (53.6%). The mean age of the cohort was 32.05 ± 12.11 years, and the mean BMI was 22.83 ± 5.91 kg/m^2^. The most common RACHS-1 category was Category 1 (51.8%), followed by Category 2 (30.1%) and Category 3 (18.1%). Of the 59% of patients with comorbid conditions, 36.7% had obesity, followed by 14.5% with hypertension, and 6% with anemia. Only 6% of patients had undergone a previous operation for their CHD. The demographic and preoperative characteristics have been summarized in Table 1.

**Table 1:** Demographics and preoperative characteristics ICU: intensive care unit; BMI: body mass index; BSA: body surface area; RACHS-1: Risk Adjustment for Congenital Heart Surgery 1; CHD: congenital heart disease; LV: left ventricle.

Variables	Total	Prolonged length of ICU stay		p-value
		Yes	No	
	N = 166 (100%)	N = 43 (100%)	N = 123 (100%)	
Gender: Male	89 (53.6)	27 (62.8)	62 (50.4)	0.161
Gender: Female	77 (46.4)	16 (37.2)	61 (49.6)	
Age (years)	32.05 ± 12.11	31.88 ± 11.77	32.11 ± 12.27	0.959
BMI (kg/m^2^)	22.83 ± 5.91	22.43 ± 6.87	22.97 ± 5.56	0.098
BSA (m^2^)	0.22 ± 0.02	1.59 ± 0.19	1.61 ± 0.22	0.099
Previous surgery	10 (6.0)	3 (7.0)	7 (5.7)	0.720
RACHS-1: Category 1	86 (51.8)	11 (25.6)	75 (61.0)	< 0.001
RACHS-1: Category 2	50 (30.1)	14 (32.6)	36 (29.3)	
RACHS-1: Category 3	30 (18.1)	18 (41.9)	12 (9.8)	
CHD type: Biventricular repair	162 (97.5)	40 (93.0)	122 (99.2)	0.054
CHD type: Univentricular repair	4 (2.5)	3 (7.0)	1 (0.8)	
Creatinine (mg/dL)	0.75 ± 0.24	0.81 ± 0.32	0.73 ± 0.20	0.123
Creatinine clearance (mL/min)	116.53 ± 41.20	110.98 ± 44.04	118.47 ± 40.17	0.796
Preoperative cyanosis	31 (18.67)	11 (25.6)	20 (16.3)	0.177
Preoperative hemoglobin (g/dL)	13.86 ± 3.04	13.90 ± 3.54	13.85 ± 2.86	0.191
Preoperative LV function: Normal	139 (83.7)	31 (72.1)	108 (87.8)	0.034
Preoperative LV function: Mild dysfunction	19 (11.4)	7 (16.3)	12 (9.8)	
Preoperative LV function: Moderate dysfunction	8 (4.8)	5 (11.6)	3 (2.4)	
Any comorbid disease	98 (59.0)	28 (65.1)	70 (56.9)	0.346
Obesity	61 (36.7)	15 (34.9)	46 (37.4)	0.768
Hypertension	24 (14.5)	5 (11.6)	19 (15.4)	0.540
Preoperative anemia	10 (6)	2 (4.7)	8 (6.5)	> 0.999
Preoperative arrhythmia	7 (4.2)	2 (4.7)	5 (4.1)	> 0.999
Diabetes mellitus	7 (4.2)	1 (2.3)	6 (4.9)	0.678

Among the sample, 43 patients were categorized as the prolonged ICU group, while the rest were classified as the non-prolonged ICU stay group. The mean ICU stay in the prolonged and non-prolonged ICU groups was 10.40 ± 4.99 and 3.96 ± 1.55 days, respectively. The prolonged ICU group had a significantly higher percentage of patients in RACHS-1 Category 3 (41.9% vs 9.8%; p < 0.001). Moreover, the prolonged ICU stay group had significantly higher mild and moderate preoperative left ventricular dysfunction (16.3% and 11.6%, respectively) than the non-prolonged ICU stay group (9.8% and 2.4%, respectively; p = 0.034).

Intraoperative and postoperative characteristics

The commonest operation performed was ASD closure (42.2%), followed by TOF repair (13.9%), VSD closure (13.3%), and AV repair (11.4%). The remaining patients (n = 32; 19.3%) had undergone mitral valve repair (n = 10; 6%), pulmonary valve replacement (n = 3; 1.8%), aortic coarctation repair on cardiopulmonary bypass (n = 3; 1.8%), tricuspid valve replacement for Ebstein anomaly (n = 2; 1.2%), Fontan operation (n = 2; 1.2%), patent ductus arteriosus closure (n = 2; 1.2%), right ventricular infundibulectomy (n = 2; 1.2%), partially anomalous pulmonary venous connection surgery (n = 1; 0.6%), repair of double-outlet right ventricle (n = 1; 0.6%), and surgery for combination lesions (n = 6; 3.6%). Postoperative complications were seen in 38.6% of patients, with acute kidney injury (AKI) being the most common (29.5%). There was no mortality in our patient cohort, and only one patient in the non-pediatric intensive care unit (PICU) group required pacemaker implantation.

The prolonged ICU stay group had a significantly higher cardiopulmonary bypass time (CPBT) (147.09 ± 69.67 vs 93.22 ± 55.94 mins; p < 0.001) and aortic cross-clamp time (XCT) (100.67 ± 55.70 vs 63.80 ± 42.77 mins; p < 0.001). Furthermore, 55.8% of patients in the prolonged ICU group needed intraoperative blood transfusion as opposed to 35.8% in the non-prolonged ICU stay group (p = 0.021). The mean intraoperative inotrope score was also significantly higher in the prolonged ICU than the non-prolonged ICU stay group (9.07 ± 9.05 vs 4.86 ± 4.67; p = 0.005). Postoperatively, serum creatinine levels on days 1, 2, and 3 were significantly higher in the prolonged ICU group (p = 0.003, 0.006, and 0.002 respectively). The prolonged ICU group had a significantly higher AKI occurrence (51.2% vs 22.0%; p < 0.001), longer duration of ventilation (3.00 ± 3.36 vs 1.29 ± 0.74 days; p = 0.002), and a longer length of hospital stay (12.77 ± 8.33 vs 6.50 ± 1.65 days; p < 0.001) (Table 2).

**Table 2:** Intraoperative and postoperative characteristics ICU: intensive care unit; ASD: atrial septal defect; TOF: tetralogy of Fallot; VSD: ventricular septal defect; AVR: aortic valve repair; CPBT: cardiopulmonary bypass time; XCT: aortic cross-clamp time; AKI: acute kidney injury.

Variables	Total	Prolonged length of ICU stay		p-value
		Yes	No	
	N = 166 (100%)	N = 43 (100%)	N = 123 (100%)	
Surgery: ASD closure	70 (42.2)	7 (16.3)	63 (51.2)	< 0.001
Surgery: TOF repair	23 (13.9)	7 (16.3)	16 (13.0)	
Surgery: VSD closure	22 (13.3)	6 (14.0)	16 (13.0)	
Surgery: AVR repair	19 (11.4)	7 (16.3)	12 (9.8)	
Surgery: Others	32 (19.3)	16 (37.1)	16 (13.0)	
CPBT (mins)	107.18 ± 64.10	147.09 ± 69.67	93.22 ± 55.94	< 0.001
XCT (mins)	73.36 ± 49.04	100.67 ± 55.70	63.80 ± 42.77	< 0.001
Intraoperative need for blood transfusion	68 (41.0)	24 (55.8)	44 (35.8)	0.021
Intraoperative inotrope score	5.95 ± 6.36	9.07 ± 9.05	4.86 ± 4.67	0.005
Any postoperative complication	64 (38.6)	21 (48.8)	43 (35.0)	0.108
AKI developed	49 (29.5)	22 (51.2)	27 (22.0)	< 0.001
Any arrhythmia	13 (7.8)	3 (7.0)	10 (8.1)	> 0.999
Surgical site bleeding	9 (5.4)	3 (7.0)	6 (4.9)	0.697
Surgical site infection	2 (1.2)	0 (0.0)	2 (1.6)	> 0.999
Pneumonia	4 (2.4)	2 (4.7)	2 (1.6)	0.276
Postoperative ventilation duration (days)	1.73 ± 1.96	3.00 ± 3.36	1.29 ± 0.74	0.002
Any postoperative procedure	12 (7.2)	6 (14.0)	6 (4.9)	0.080
Postoperative chest reopened	8 (4.8)	4 (9.3)	4 (3.3)	0.207
Other postoperative procedures	5 (3.0)	3 (7.0)	2 (1.6)	0.110
Postoperative reintubation	4 (2.4)	2 (4.7)	2 (1.6)	0.276
Length of hospital stay (days)	8.12 ± 5.22	12.77 ± 8.33	6.50 ± 1.65	< 0.001

Among patients with ASD closure, only 10.0% of patients (seven of 70) required prolonged ICU stay. In this subgroup, patients with postoperative complications vs no postoperative complications (27.0% vs 3.0%; p = 0.004) and prolonged mechanical ventilation (3.86 ± 2.48 vs 1.22 ± 0.73 days) were more likely to require prolonged ICU care.

Logistic regression

On multivariable logistic regression adjusted for age, gender, and RACHS-1 category, prolonged ICU stay was associated with intraoperative inotrope score (OR 1.098; 95%CI: 1.032-1.169), CPBT (OR 1.013; 95%CI: 1.005-1.021), XCT (OR 1.015; 95%CI: 1.005-1.025), duration of mechanical ventilation (OR 1.896; 95%CI: 1.339-2.684), and postoperative AKI (OR 2.847; 95%CI: 1.233-6.571) (Table 3).

**Table 3:** Univariate and multivariable regression analysis for prolonged intensive care unit (ICU) stay Multivariable regression was adjusted for age, gender, and RACHS-1 category ICU: intensive care unit; cOR: crude odds ratio; aOR: adjusted odds ratio; BMI: body mass index; CPBT: cardiopulmonary bypass time; XCT: aortic cross-clamp time; AKI: acute kidney injury; RACHS-1: risk adjustment for congenital heart surgery 1

Variables	cOR [95% CI]	p-value	aOR [95% CI]	p-value
BMI (kg/m^2^)	0.984 [0.927-1.045]	0.606	0.982 [0.908-1.061]	0.639
Intraoperative transfusion	2.268 [1.120-4.594]	0.023	2.065 [0.917-4.649]	0.080
Intraoperative inotrope score	1.105 [1.041-1.172]	0.001	1.098 [1.032-1.169]	0.003
CPBT (mins)	1.014 [1.007-1.020]	< 0.001	1.013 [1.005-1.021]	0.001
XCT (mins)	1.016 [1.008-1.023]	< 0.001	1.015 [1.005-1.025]	0.003
Hypotension on arrival	1.472 [0.630-3.441]	0.372	1.064 [0.385-2.939]	0.904
Postoperative ventilation duration (days)	1.790 [1.314-2.437]	< 0.001	1.896 [1.339-2.684]	< 0.001
Any postoperative complication	1.776 [0.879-3.589]	0.110	1.392 [0.631-3.071]	0.413
AKI developed	3.725 [1.787-7.766]	< 0.001	2.847 [1.233-6.571]	0.014

## Discussion

In this single-center study, we investigated the association of various preoperative, intraoperative, and postoperative factors with prolonged ICU stay. On multivariable logistic regression adjusted for age, gender, and RACHS-1 category, we found several factors to be associated with prolonged ICU stay, including intraoperative ionotropic score, CPBT, XCT, post-operative ventilation duration, and AKI.

The age of patients in our study (mean: 32.05 years) is comparable to others by Kwiatkowski et al. (median: 29 years) and Karangelis et al. (mean: 36.1 years) [20,21]. However, while most patients in our study belonged to RACHS-1 Category 1 and Category 2 (51.8% and 30.1%, respectively), this is inconsistent with previous studies (Category 1: 12-29.7%; Category 2: 26.9-39%; Category 3: 42-74.4%) [22-24]. In LMICs like Pakistan, limited access to appropriate specialized care during childhood contributes to a 60% mortality rate in children with CHD [25]. Children with simpler defects are more likely to survive into adulthood, frequently undiagnosed, causing the bulk of ACHD to consist of lower RACHS-1 categories (mostly Category 1 and Category 2) [26,27].

In our study, intraoperative inotrope score was positively associated with prolonged ICU stay (OR 1.098) on multivariable logistic regression. A limited number of studies have assessed the relation between intraoperative inotrope score and outcomes in adult populations undergoing surgery for CHD. Early postoperative inotrope score has been known to predict unfavorable postoperative outcomes including longer ICU stays after cardiac surgery [28], including surgery for ACHD [29]. However, limited studies explore this association with intraoperative inotrope score. Yamazaki et al. in Japan demonstrated that a high inotrope score was associated with a longer ICU stay after ACHD surgery [30]. Thus, while our findings are supported by previous literature, additional studies are needed to explore the predictive relationship of intraoperative inotrope score during ACHD surgery with postoperative ICU stay and other clinically significant outcomes in different populations.

Other intraoperative factors associated with prolonged ICU stay on multivariable logistic regression were CPBT (OR 1.013; 95%CI 1.005-1.021) and XCT (OR 1.015, 95%CI 1.005-1.025). Both CPBT and XCT are well known to be associated with longer ICU stay after cardiac surgery across age groups [31,32], including surgery for ACHD [21]. Nevertheless, this is the first study from Pakistan to report the association of longer CPBT and XCT with prolonged ICU stay after surgery for ACHD. Postoperatively, prolonged ICU stay was associated with a longer duration of mechanical ventilation (OR 1.896; 95%CI 1.339-2.684) and development of AKI (OR 2.847, 95%CI 1.233-6.571). This relationship between development of AKI and longer ICU stay after surgery for ACHD has been described previously, and AKI itself may be predicted by the complexity of ACHD surgery and excess intraoperative blood loss [33]. Similarly, the requirement of prolonged mechanical ventilation is associated with longer postoperative ICU stay, as has been demonstrated in a pediatric population [34]. Interestingly, prolonged mechanical ventilation is also associated with higher inotrope score [34], and longer CPBT and XCT [35], corroborating the interplay between these variables in prolonging ICU stay as shown in our study.

Keeping in mind the effect of longer ICU stays on the outcomes of patient populations and hospital resource consumption [4-8], our findings are of relevance to resource-constrained settings. Pakistan has a severe shortage of ICU beds (1.5 per 100,000 population), similar to other LMICs like Bangladesh (0.7), India (2.3), and Sri Lanka (2.3). This contrasts with high-income countries in the region, such as Taiwan (28.5) and Saudi Arabia (22.8) [36]. This emphasizes the importance of our findings. The duration of ICU stay may be actively minimized through measures such as the judicious use of intraoperative inotropes and shorter operative durations during surgery for ACHD as well as the anticipation and prompt management of postoperative complications. Involvement of multidisciplinary surgical teams in LMICs is warranted to identify factors contributing to prolonged CPBT, XCT, ventilation duration, excessive inotrope usage, and development of AKI postoperatively, particularly in non-complex cases. Such collaborative efforts can inform evidence-based quality improvement initiatives aimed at reducing ICU stay among patients undergoing surgery for ACHD. Furthermore, development and implementation of on-table extubation and fast-track ICU shift-down protocols in LMICs like ours is another area necessitating future research [11,37].

There were several limitations to this study. The sample size used was relatively small due to the limited volume of adult patients in the institution who were operated on for ACHD. All the patients belonged to categories 1, 2, and 3. No patients underwent surgeries classified as RACHS-1 categories 4, 5, and 6, and thus our findings were not adjusted for higher RACHS-1 categories. Moreover, with a low rate of postoperative complications due to a majority of simple surgeries, and no mortality, we were unable to fully explore the association of prolonged ICU stay with postoperative complications and mortality.

## Conclusions

Prolonged ICU stay may be associated with intraoperative factors, such as intraoperative ionotropic score, CPBT, and XCT, in addition to postoperative factors, such as postoperative ventilation duration and AKI. Surgeons managing ACHD in Pakistan must strive for shorter operative durations and the judicious use of inotropes during surgery, and anticipate postoperative complications such as AKI, to minimize ICU stay. Such efforts would ensure evidence-based resource utilization while also alleviating the risk of morbidity associated with prolonged length of stay.
